# Personal Protection of Permethrin-Treated Clothing against *Aedes aegypti*, the Vector of Dengue and Zika Virus, in the Laboratory

**DOI:** 10.1371/journal.pone.0152805

**Published:** 2016-05-17

**Authors:** James Orsborne, Sarah DeRaedt Banks, Adam Hendy, Salvador A. Gezan, Harparkash Kaur, Annelies Wilder-Smith, Steve W. Lindsay, James G. Logan

**Affiliations:** 1 Department of Disease Control, London School of Hygiene and Tropical Medicine, London, United Kingdom; 2 SFRC, University of Florida, Gainesville, Florida, United States of America; 3 Department of Global Health and Epidemiology, Umea University, Umea, Sweden; 4 Lee Kong Chian School of Medicine, Nanyang Technological University, Singapore, Singapore; 5 School of Biological and Biomedical Sciences, Durham University, Durham, United Kingdom; 6 arctec, London School of Hygiene and Tropical Medicine, London, United Kingdom; United States Department of Agriculture, Beltsville Agricultural Research Center, UNITED STATES

## Abstract

**Background:**

The dengue and Zika viruses are primarily transmitted by *Aedes aegypti* mosquitoes, which are most active during day light hours and feed both in and outside of the household. Personal protection technologies such as insecticide-treated clothing could provide individual protection. Here we assessed the efficacy of permethrin-treated clothing on personal protection in the laboratory.

**Methods:**

The effect of washing on treated clothing, skin coverage and protection against resistant and susceptible *Ae*. *aegypti* was assessed using modified WHO arm-in-cage assays. Coverage was further assessed using free-flight room tests to investigate the protective efficacy of unwashed factory-dipped permethrin-treated clothing. Clothing was worn as full coverage (long sleeves and trousers) and partial coverage (short sleeves and shorts). Residual permethrin on the skin and its effect on mosquitoes was measured using modified WHO cone assays and quantified using high-pressure liquid chromatography (HPLC) analysis.

**Results:**

In the arm-in-cage assays, unwashed clothing reduced landing by 58.9% (95% CI 49.2–66.9) and biting by 28.5% (95% CI 22.5–34.0), but reduced to 18.5% (95% CI 14.7–22.3) and 11.1% (95% CI 8.5–13.8) respectively after 10 washes. Landing and biting for resistant and susceptible strains was not significantly different (p<0.05). In free-flight room tests, full coverage treated clothing reduced landing by 24.3% (95% CI 17.4–31.7) and biting by 91% (95% CI 82.2–95.9) with partial coverage reducing landing and biting by 26.4% (95% CI 20.3–31.2) and 49.3% (95% CI 42.1–59.1) respectively with coverage type having no significant difference on landing (p<0.05). Residual permethrin was present on the skin in low amounts (0.0041mg/cm^2^), but still produced a KD of >80% one hour after wearing treated clothing.

**Conclusion:**

Whilst partially covering the body with permethrin-treated clothing provided some protection against biting, wearing treated clothing with long sleeves and trousers provided the highest form of protection. Washing treated clothing dramatically reduced protection provided. Permethrin-treated clothing could provide protection to individuals from *Ae*. *aegypti* that show permethrin resistance. Additionally, it could continue to provide protection even after the clothing has been worn. Field trials are urgently needed to determine whether clothing can protect against dengue and Zika.

## Introduction

Dengue fever is a threat to most of the tropics and sub-tropics [1]. The World Health Organisation (WHO) estimates that 50–100 million infections and 20,000 deaths occur annually due to dengue fever and a 30 fold increase in incidence of dengue globally in the past 50 years [1, 2]. Recently, the Zika virus swept through South and Central America with more than one million estimated cases along with a possible link to children born with microcephaly [3–5].

The major vector of both viruses is the *Aedes aegypti* mosquito. This day-biting mosquito is closely associated with urban environments in the tropics and subtropics, where they breed in a wide variety of man-made containers and small natural water bodies [6–10]. There is no specific antiviral therapy for dengue fever or Zika. Clinical management of dengue depends upon prompt institution of fluid replacement therapy [11, 12] and despite significant progress in dengue vaccines development. There is still no licenced vaccine available [13, 14]. Currently Zika case management is based on pain relief and fever reduction. There is also no licenced vaccine available [15]. Therefore, vector control is the main method of reducing the burden of these diseases [1, 16]. Primary strategies include the use of larval source management, indoor residual spraying (IRS) and ultra-low volume (ULV) spraying of insecticides to reduce the population of *Aedes* mosquitoes [1, 17], but protective efficacy is highly variable [18–21]. Wolbachia and genetic modification strategies for *Aedes* control have shown promise, but are still in the development stage and require more data before being implemented on a large scale [22]. Therefore, there is a need for effective, targeted measures for the control of *Aedes* vectors [23].

Personal protection technologies, such as insecticide-treated clothing, have been used by the military and other commercial companies to protect workers from biting arthropods for many years [24]. Permethrin-treated clothing has been shown to reduce bites from disease vectors such as ticks and mosquitoes [25, 26] as well as reduce the prevalence of some vector borne diseases such as leishmaniasis and malaria [27–29]. As the clothing can be worn when an individual is outside of their home during the day, it has the potential to provide long-term protection from day biting mosquitoes.

When measuring efficacy of insecticide-treated clothing, previous studies have primarily focused on knock down and mortality as the primary outcome rather than actual protection against biting, which is arguably the most important outcome for individual protection against infection from a mosquito bite [24]. Often these tests are done in small scale laboratory studies and do not take account of “real-life” scenarios such as partial coverage of the body with clothing (e.g. short sleeves and shorts) and the presence of insecticide-resistant mosquitoes. In our study, we assess the effects of skin coverage, mosquito resistant status and effects of washing on duration of protection provided by permethrin-treated clothing using standardised WHO arm-in-cage assays. We further investigated the effects skin coverage of permethrin-treated clothing under more realistic situations, utilising specialised free-flight rooms.

## Methods

### Clothing

Long sleeved, 100% cotton beige shirts, black trousers and shorts, were used in this study (Insect Shield, Greenboro, US). All clothing was treated with permethrin using a polymer-coating technique (542 mg/m^2^) by Insect Shield (Insect Shield, Greenboro, US). Control clothing consisted of identical shirts and trousers that had not been treated. The treated clothing was odourless and could not be distinguished from the untreated clothing. For full coverage, the participant wore a long-sleeved shirt and long trousers. For partial coverage, sleeves were rolled up to above the elbow and shorts were worn.

### Washing method

An automatic washing machine (Hotpoint, model WMSL 521P, Peterborough, UK) was used to wash the clothes. Separate washing machines were used for the control and treated clothing. The machines were set for an eco-cycle with a water temperature of 30°C and a spin speed of 800rpm. 25ml of unscented washing powder (pH 10) (Violet’s Homescents, Northumberland, UK) was used for every wash, which used 59L of water with clothing weighing approximately 140 grams. After each wash, the clothing was hung and left to dry at 30°C in designated control and treated free-standing drying cabinets (LTE Scientific, fan extracted model). An empty machine wash was done between each clothing wash, so residual insecticide was removed from the washing machine drum.

### Mosquitoes

Permethrin-susceptible *Ae*. *aegypti*, (originally from West Africa, colonised in 1926 with field additions in 1976) were obtained from a reference strain held at the London School of Hygiene and Tropical Medicine (LSHTM, London, UK) and a permethrin-resistant strain obtained from the Liverpool School of Tropical Medicine (Liverpool, UK). The resistant strain (Cayman) originally from the Grand Cayman Islands has been reared at the Liverpool School of Tropical Medicine (Liverpool, UK) since 2008 under standard lab conditions (26°C, 80% RH and a 12: 12 hours light: dark cycle) with resistance maintained (Pyrethroid, carbamate & DDT resistant. Deltamethrin LC50 9.29 ug/ml, *kdr* 0.7). All mosquitoes were reared and housed at the LSHTM under optimal environmental conditions of 25°C ± 2°C and 80% RH with a 12:12 hour photoperiod. Female mosquitoes between the ages of a 3–7 days were used. All mosquitoes had never been blood-fed but had access to sugar before experiments were performed.

### Arm-in-cage experiments

The following material was tested in the arm-in-cage experiments: 1) a forearm covered fully by the treated clothing (FCT), 2) a forearm partially covered with treated material (PCT; the material covered half of the length of the forearm) 3) full coverage untreated control (FCC; a forearm fully covered with untreated material), 4) partial coverage untreated control (PCC; a forearm partially covered with untreated material) and 5) a bare arm control (BA).

Thirty 5–7 day old female mosquitoes were used for each arm-in-cage experiment. Mosquitoes were transferred using a mouth aspirator fitted with a high-efficiency particulate arrestance (HEPA) filter and left to acclimatise for one hour before testing commenced. Biting pressure was checked by placing one bare arm with a glove on the hand into the cage for up to 30 seconds. The cage was refreshed with new mosquitoes if fewer than 10 mosquitoes landed and probed in the 30 second time period and this was repeated when necessary until a satisfactory biting pressure was achieved. The test material was then wrapped tightly on the participants forearm and the arm inserted into the cage for 90 seconds. After the 90 second time period, the number of mosquitoes probing on the arm was counted, and 2 minutes after the end of the test, the number of visible bites (wheals) on the arm were counted. Before and after each test, the forearm was washed with fragrance free soap (Boots, UK), water and wiped down with 75% ethanol.

Two experiments were done using the arm-in-cage assay (see below). Both experiments were performed following WHOPES [30] guidelines with some modifications to allow testing of clothing rather than a conventional topical repellent. Tests were performed every hour with all treatments tested once in the same day using a Latin square design. Any bite reactions still present from the previous tests were marked with a colored indelible pen to avoid miscounting in subsequent experiments. Ten replicates (equating to 300 mosquitoes per treatment) were performed using one participant.

### Experiment 1: Washing effect

The forearm of a single participant was wrapped in control clothing (unwashed), or treated clothing that had been unwashed or machine washed 1, 5 or 10 times. Each of the treatment types (FCT and PCT) were compared against untreated control material (FCC and PCC) and a bare arm control (BA).

### Experiment 2: Resistant versus susceptible

Two strains of *Ae*. *aegypti* mosquito were tested: a permethrin-susceptible strain and permethrin-resistant strain. The forearm of a single participant was wrapped in either untreated or treated clothing (FCC, PCC or FCT, PCT, respectively) and the arm was placed in a cage containing either resistant or susceptible mosquitoes. Both full and partial coverage (FCT and PCT) were tested with each of the treatment types and compared against untreated control material (FCC or PCC) and a bare arm (BA) control.

### Free-flight room experiments

**Fig 1:**
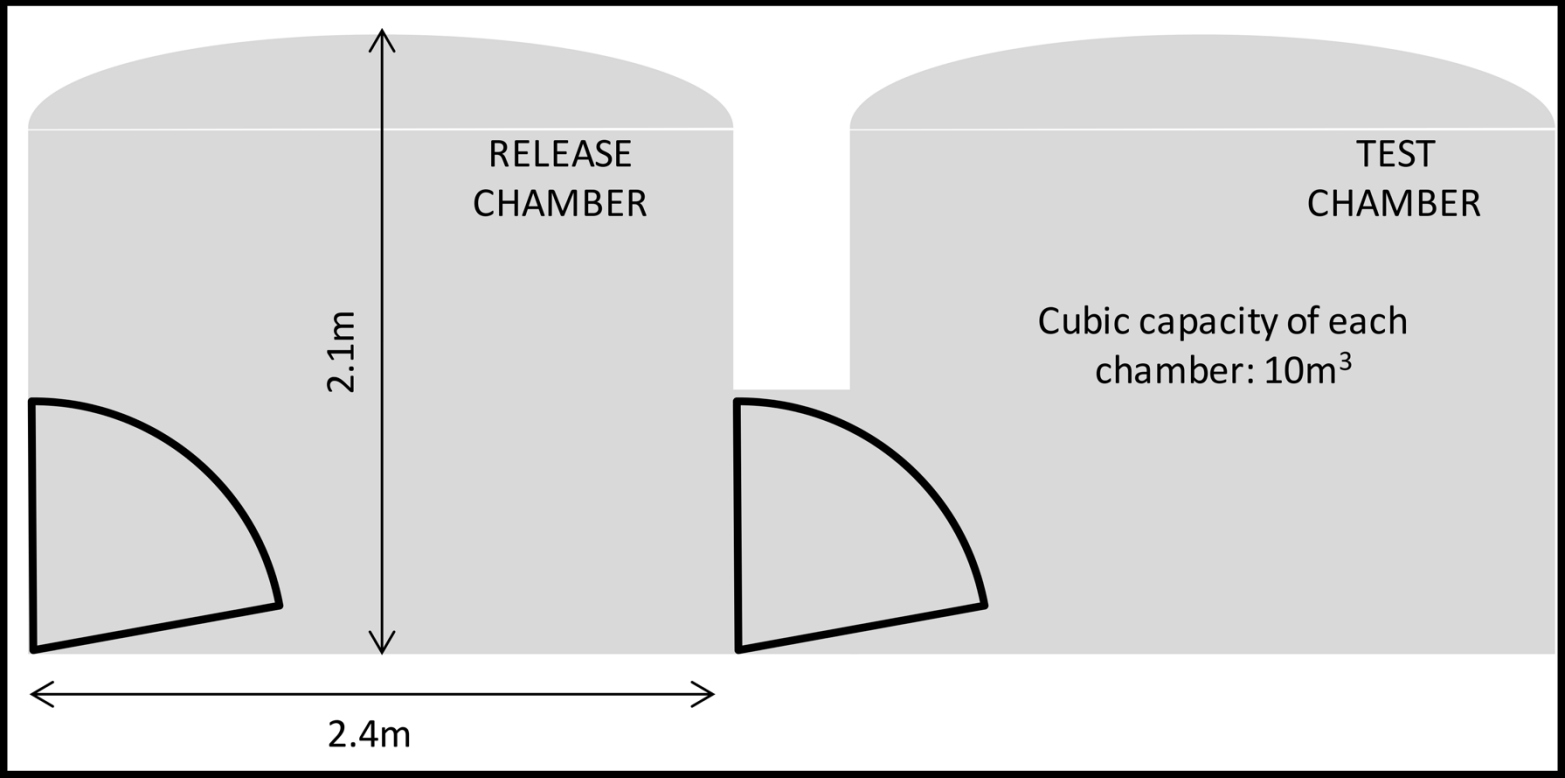
Free-flight room schematic layout.

Two white, tiled, sealed test rooms (10m^3^, 25°C ± 2°C and 75 ± 10% RH), connected by a single door, were used to test the clothing for all experiments (Fig 1). The free-flight room provided semi-natural conditions where mosquitoes were allowed to fly, rest and blood-feed freely. Thirty mosquitoes were released in the release chamber while a participant sat in the test chamber wearing the clothing, with bare feet and hands exposed and with head covered using a head net. Each experiment in the free-flight room was run for 15 minutes. Two experiments were run. In the first experiment, the participants recorded the number of mosquitoes that landed on their exposed skin. The mosquitoes were removed by a mouth aspirator as soon as the mosquitoes probed the skin so they were not double counted. In the second experiment, the number of mosquitoes were not removed upon probing, but were allowed to fully blood feed. The number of mosquitoes that had blood fed were then counted at the end of the experiment.

After each experiment, all mosquitoes in the release chamber and the test chamber were collected using a mouth aspirator. The free-flight room was wiped down with 75% ethanol and force-ventilated for 30 minutes between tests to remove any potential insecticide or human volatiles. The participants washed all limbs with fragrance-free soap (Boots, UK), water and 75% ethanol after each test. New mosquitoes were used for each test with all treatments being tested once in the same day. The 4 treatments (FCT, PCT, FCC and PCC) and a negative control of PCC clothing were allocated according to a Latin square design blocked across 5 hours. The same participant was used to test all treatments in a single day. In total, twelve participants (6 male and 6 female) were used across the two experiments, with each experiment consisting of 6 participants with a 1:1 male/female ratio.

### Residual efficacy on skin

**Fig 2:**
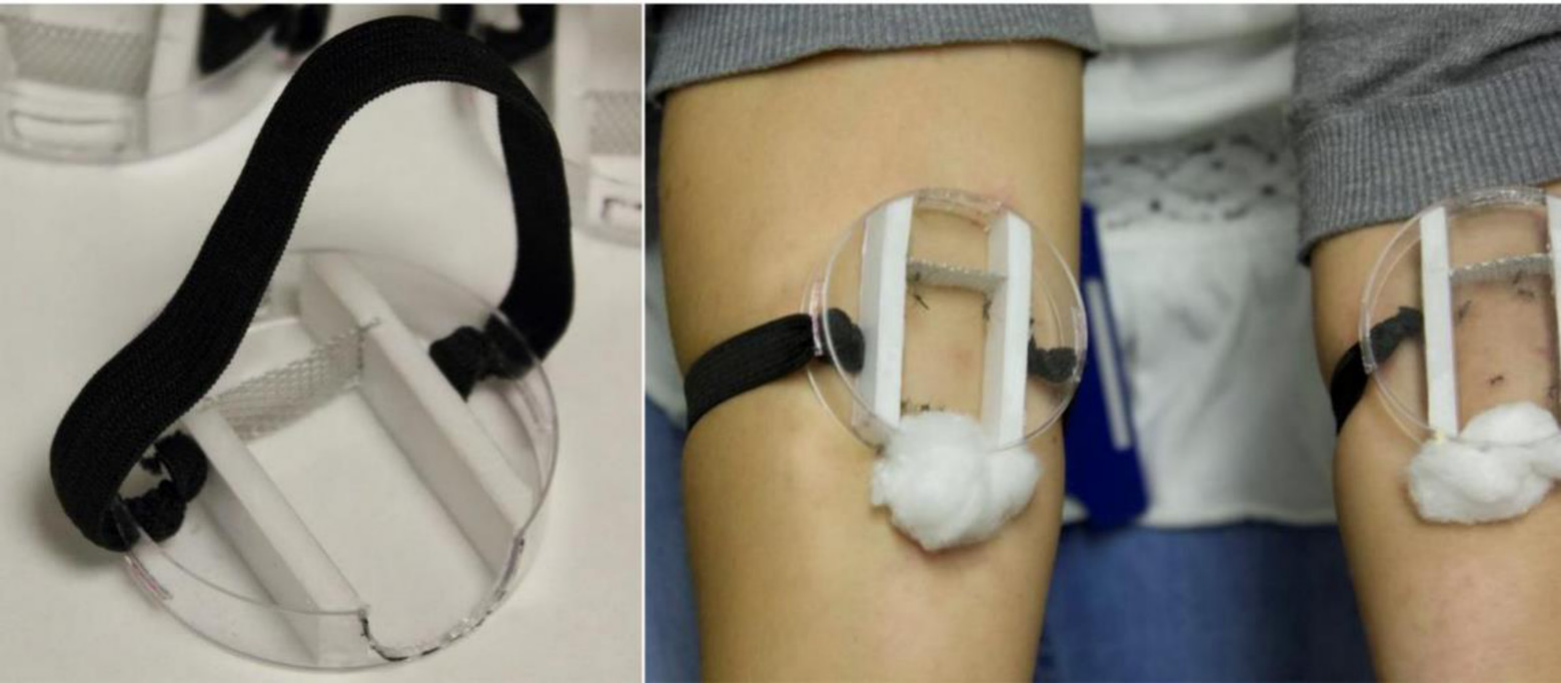
Adapted mosquito holding chamber for skin residual efficacy experiments.

The purpose of this experiment was to establish whether permethrin was transferred from treated clothing onto the skin surface and whether it accumulated in a sufficient amount to affect mosquito behaviour, KD or mortality. Ten participants tested a permethrin-treated and an untreated sleeve. Participant and investigator were blinded to the treatments. Participants wore the sleeves for up to 7 hours per day for 5 days, after which time the sleeves were removed. Five 7–8 day old non-blood fed mosquitoes were aspirated into an exposure chamber (Fig 2) attached to the participant’s forearm. One chamber was placed onto the forearm that had been exposed to the treated sleeve and one chamber attached to the unexposed arm and mosquitoes were inserted into the chamber. After 3 minutes mosquitoes were removed, placed in holding cups and given 10% glucose in a humidity recovery chamber (25 ± 2°C and 75 ± 10 RH%). This was repeated with new mosquitoes one hour after the first exposure. High-Performance Liquid Chromatography (HPLC) was used to analyse skin swabs taken immediately after wearing the clothing and one hour after to quantify the presence of permethrin. A 3cm x 3cm template was placed on the arm of a participant, and ethanol-soaked cotton buds were used to swab the area at both time points. Swabs were allowed to dry, wrapped in foil and then sealed in centrifuge tubes until processed.

### High Pressure Liquid Chromatography (HPLC)

Analyses were carried out using a Dionex Ultimate 3000 range of equipment and software (Camberly, Surrey, UK). Samples were separated on an Acclaim^®^ C_18_ 120 Ǻ (250 x 4.6 mm, Dionex, UK) column eluting with water/acetonitrile (90:10%; v/v) at a flow rate of 2 ml/minute and passed through the photodiode array detector (PDA-100, Dionex) set at 275 nm. The authenticity of the detected peaks was determined by comparison of retention time, spectral extraction at 275 nm and spiking the sample with commercially available standard of the insecticide. A calibration curve of insecticide was generated by Chromeleon (Dionex software) using known amounts of the standard (0–0.4 ug/ml) in acetonitrile injected onto the column. From this curve the amount of insecticide in the matrix was calculated.

## Statistical Analysis

### Arm-in-cage experiments

A generalised linear model (GLM) was fitted based on a binomial distribution with a logic link. Factors such as temperature, humidity and time of day were included but only factors which were shown to have a significant effect (α = 0.05) were kept in the final model. Comparisons between predicted means for each treatment type were performed using a least significant difference (LSD) post-hoc test with a significance level of 5%. The binary responses for cone assays included number of mosquitoes in the 15 minute KD, 1 hour KD, and 24 hour mortality as well as number of biting and landing mosquitoes. All models were fitted using PROC GLIMMIX implemented in SAS v. 9.3 software [31].

### Free-flight room experiments

A generalized linear model (GLM) was fitted based on a binomial distribution with a logit link (i.e. logistic regression). The effects of time, participant and fabric type were included in the model. The inclusion of the covariates of temperature and humidity were also evaluated (α = 0.05), but they were only kept in the final model if shown to have a significant effect. Comparisons between predicted means for blood feeding, landing as well as knockdown (KD) and mortality for each treatment type were performed using a LSD post-hoc test with a significance level of 5%. Statistical analyses were done using IBM SPSS software [32].

### Residual efficacy on skin experiments

A repeated measures analysis of variance (ANOVA) was performed including 3 minute KD, 1 hour KD, and 24 hour mortality at both time points (immediately after wearing and 1 hour after wearing treated clothing). Covariates for humidity, time of day and participant were also evaluated but only kept in the final model if they had a significant effect (α = 0.05). A comparison between predicted means for 3 minute KD, 1 hour KD and 24 hour mortality at both time points was performed using a LSD post-hoc test with significance set at 5%. Statistical analysis was performed using IBM SPSS software [32].

### Protection

For both free-flight room and arm-in-cage experiments, protection was defined by two components: 1) protection against landing, and 2) protection against biting and/or blood feeding. Protection was determined by expressing the number of mosquitoes landing, probing or blood fed for each treatment as a percentage of the number of mosquitoes landing on the controls. For example, the formula to determine the protection of an arm with FCT in comparison to an arm with the control FCC is: Protection (%) = 100× [(FCC–FCT)/FCC]. Similar expressions were used for other treatments.

## Ethical approvals

This study was approved by the London School of Hygiene and Tropical Medicine Ethics committee (reference numbers 6074, 7659 and 011/445). All participants were adults between the ages of 18–65 and provided written informed consent before taking part in this study. All participants were pre-screened for a visible bite reaction before taking part in the study.

## Results

### Arm-in-cage experiments

#### Washing effect

**Fig 3:**
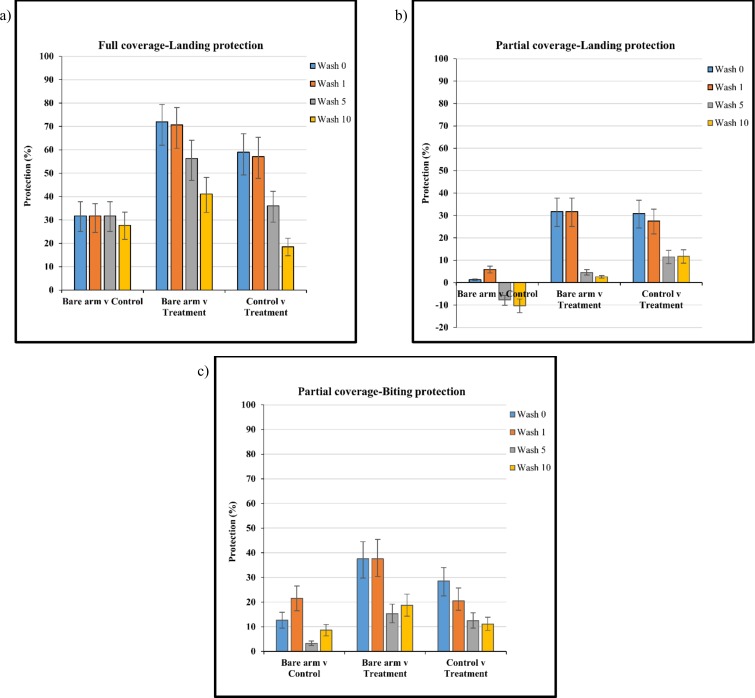
Protection (%) against (a) landing, full coverage treated (FCT) material, (b) landing, partial coverage treated (PCT) material and (c) biting, partial coverage treated (PCT) material when washed 0, 1, 5 and 10 times. Clothing protection was evaluated through comparison with the bare arm control and untreated control material. Whiskers correspond to 95% confidence intervals.

Landing protection provided by treated clothing decreased with increasing number of washes (Fig 3A, 3B and 3C). Unwashed clothing and clothing washed once showed significantly greater protection when compared to the bare arm control (p<0.001) with no difference in landing after 5 or 10 washes. FCT provided the greatest protection compared to partial coverage (p = 0.0001) with 58.9% (95% CI 49.2–66.9) protection for unwashed clothing and 18.5% (95% CI 14.7–22.3) protection after 10 washes (Fig 3A). PCC provided no significant protection against landing when compared to a bare arm (p = 0.6776). PCT clothing provided 30.9% (95% CI 24.4–36.8) landing protection but decreased to 11.7% (95% CI 8.7–14.6) after 10 washes (Fig 3B).

Both FCT and FCC materials provided a greater than 97% bite protection with washing having no significant effect. Data are not shown. PCT material reduced biting significantly compared to the control across all wash points (p = 0.0051). However, bite protection decreased as wash number increased from 28.5% (95% CI 22.5–34.0) to 11.1% (95% CI 8.52–13.8) after 10 washes (Fig 3C).

#### Resistant verses susceptible mosquitoes

**Fig 4:**
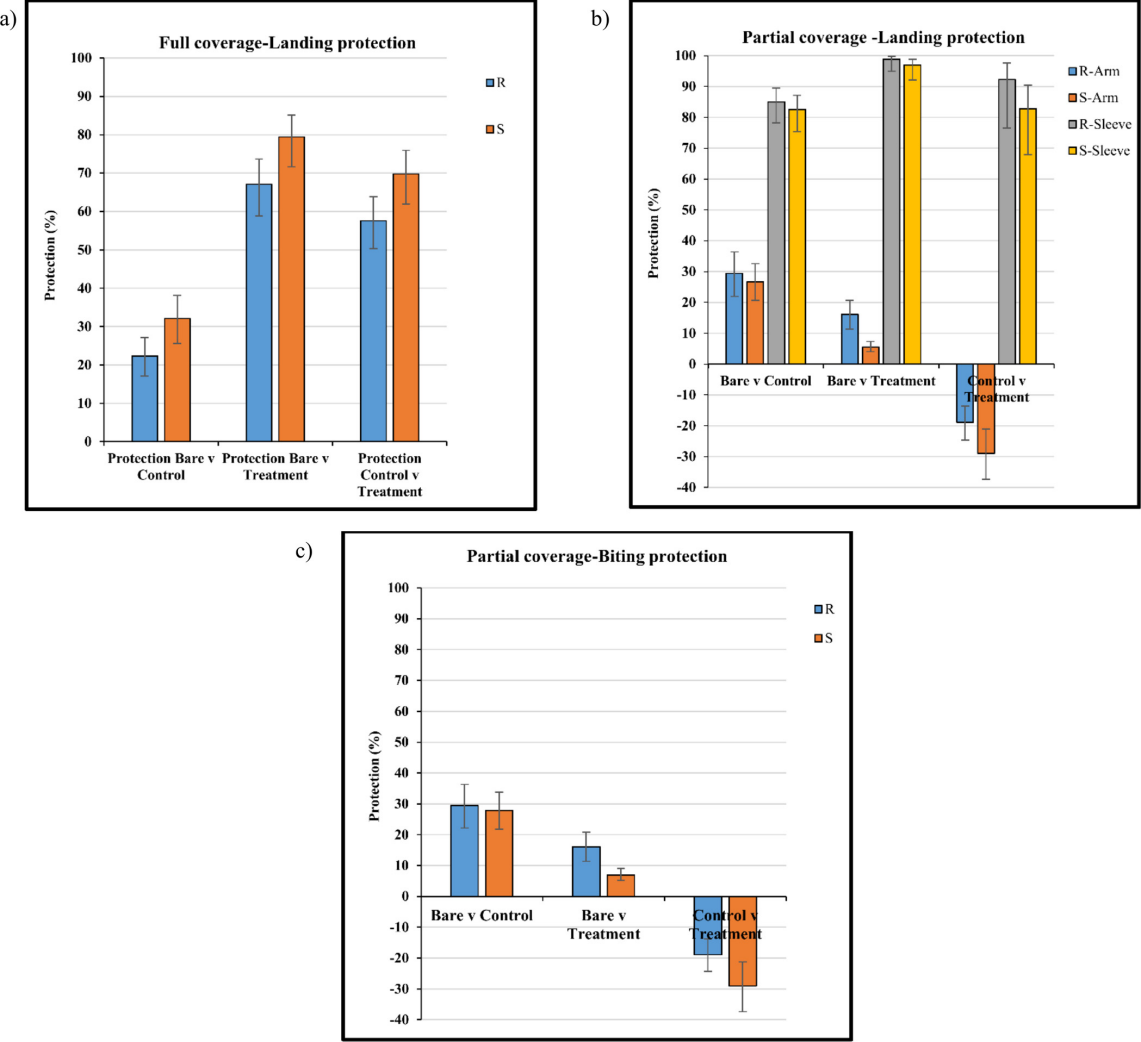
Protection (%) against (a) landing, full coverage treated (FCT) material, (b) landing, partial coverage treated (PCT) material and (c) biting, partial coverage treated (PCT) material with permethrin resistant (R) and susceptible (S) mosquito strains. Clothing protection was evaluated through comparison with the bare arm control and untreated control material. Whiskers correspond to 95% confidence intervals.

With full coverage there was no difference in landing between susceptible and resistant mosquito strains across all comparisons, with a landing protection of 57.6% (95% CI 50.3–63.8) and 69.7% (95% CI 61.9–75.9) for resistant and susceptible mosquito strains respectively (Fig 4A). Treated clothing gave significant landing protection when compared to the control clothing for both resistant and susceptible strains (p<0.0001).

For partial coverage, protection against landing on the uncovered portion of the arm was greatest when the arm was partially covered with untreated control clothing with 29.4% protection (95% CI 22.0–36.4) for the resistant strain and 26.7% protection (95% CI 20.7–32.5) for the susceptible strain (Fig 4B). Landing protection on the uncovered portion of the arm decreased for both resistant and susceptible strains when the arm was partially covered with treated clothing, with a negative protection of -18.9% (95% CI -13.6–24.6) and -29.0% (95% CI -21.1–37.3) (Fig 4B).

For the covered portion of the arm, the control sleeve provided 82.6% (95% CI 75.4–87.2) and 85.0% (95% CI 78.2–89.5) protection for resistance and susceptible strains when compared to the bare arm control. Treated clothing provided the greatest landing protection, with 92.3% (95% CI 76.6–97.7) for the resistant strain and 82.8% (95% CI 67.9–80.6) for the susceptible strain (Fig 4B). A significant difference was found between resistant and susceptible strains for landing on the arm only (p<0.0001) (Fig 4B).

For bite protection partial coverage of the arm with untreated control clothing provided the greatest protection on the uncovered portion of the arm of 29.4% (95% CI 22.2–36.3) for the resistant strain and 27.9% (95% CI 21.8–33.8) for the susceptible strain (Fig 4C). The treated clothing provided bite protection when compared to a bare arm (resistant strain = 16.1% (95% CI 11.4–20.8), susceptible strain = 7.0% (95% CI 5.1–9.1)) but no bite protection when compared to the control (resistant strain = -18.9% (95% CI -13.8–-24.6), susceptible strain = -29.0% (95% CI -21.1–-37.)) (Fig 4C). The covered area of the arm provided a greater than 99% bite protection for both control and treated clothing. Data are not shown.

## Free-Flight Room Experiments

**Fig 5:**
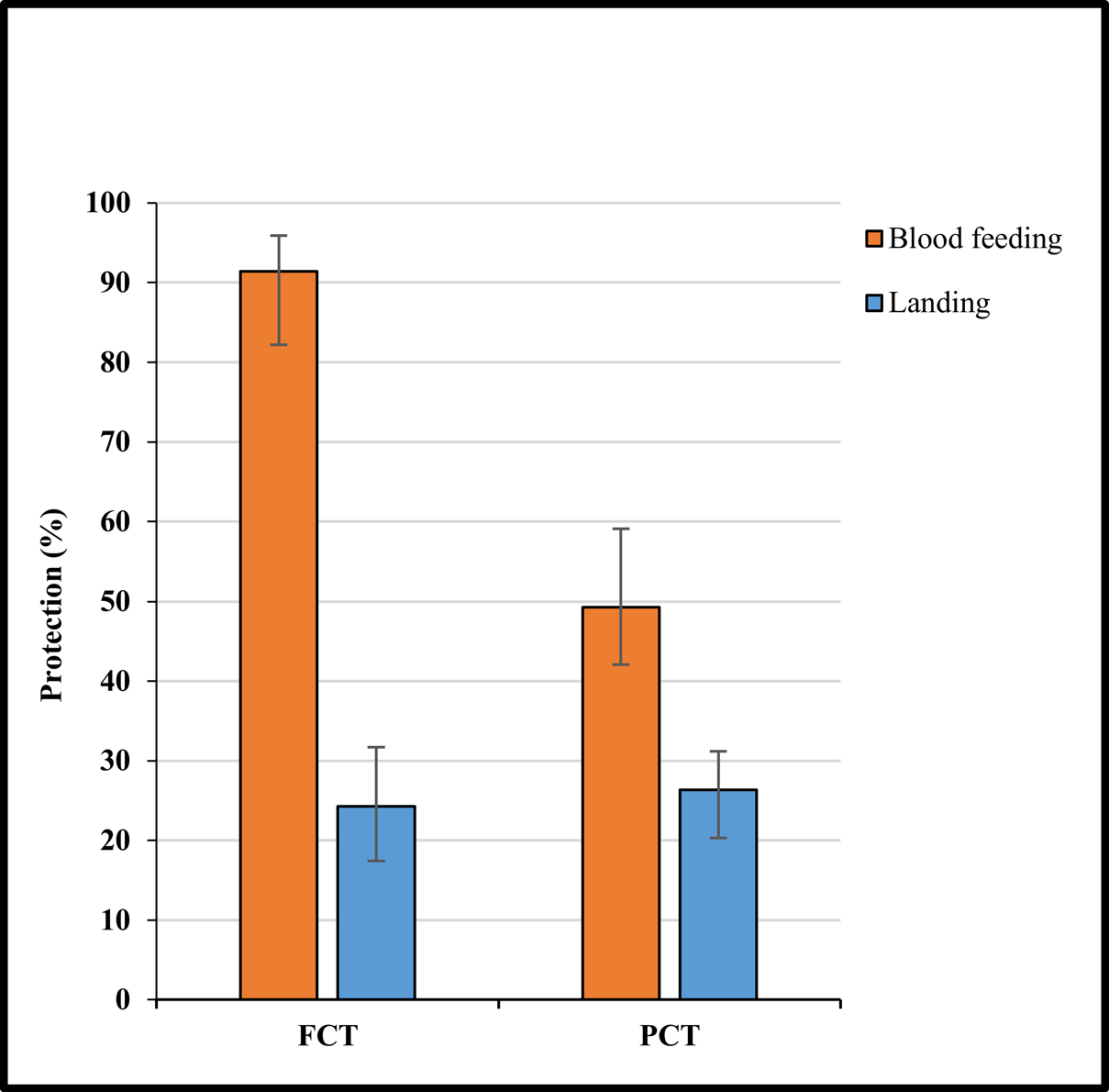
Protection (%) against blood feeding and landing when wearing partial coverage treated (PCT) or full coverage treated (FCT) clothing. Protection was calculated by comparing the treatment to partial coverage untreated clothing (PCC) in free-flight room tests. Whiskers correspond to 95% confidence intervals.

Overall, coverage had no effect on landing protection (p >0.05). FCT reduced landing by 24.3% (95% CI = 17.4–31.7) compared to its untreated control, whilst PCT provided a 26.4% (95% CI = 20.3–31.24) reduction in landing (Fig 5). Both KD and mortality were significantly greater for FCT and PCT clothing when compared to their corresponding controls (p< 0.0001). FCT clothing reduced blood feeding by 91.4% (95% CI 82.2–95.9, p >0.05) compared with its untreated control (Fig 5). The degree of protection was less with PCT (49.3%, 95% CI 42.1–59.1, p = 0.01) when compared with its untreated control (Fig 5).

**Fig 6:**
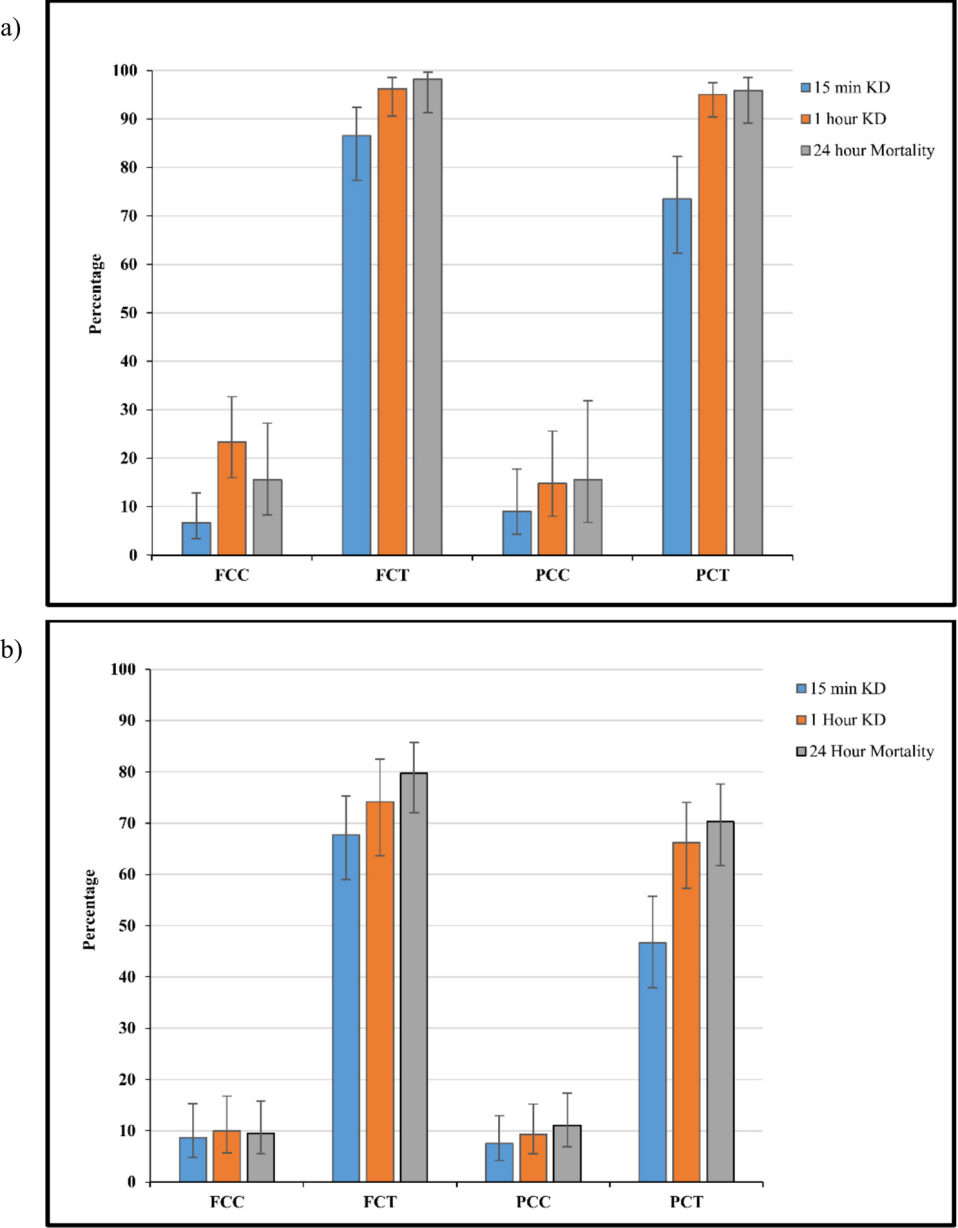
Knockdown after 15 minutes, 1 hour and mortality after 24 hours post free-flight room testing where mosquitoes were collected after (a) landing and (b) after feeding freely. Participants wore full coverage control (FCC), full coverage treated (FCT), partial coverage control (PCC) and partial coverage treated (PCT) clothing. Whiskers correspond to 95% confidence intervals.

FCT produced a KD and mortality of >80% for all time points (p<0.05) (Fig 6A). PCT KD was 73.5% (95% CI 62.3–82.3) after 15 minutes, compared to 95.1% (95% CI 90.4–97.5) at 1 hour and 95.9% (95% CI 89.1–98.5) at 24 hours post exposure (Fig 6A). For both FCT and PCT a greater than 90% KD and mortality was observed 1 hour and 24 hours post exposure with no significant difference between the two treatments (p<0.05). In the second experiment where mosquitoes were allowed to feed freely, both FCT and PCT clothing produced greater KD and mortality when compared to their corresponding controls (p< 0.0001). FCT produced a significantly greater KD at the 15 minute stage, 67.7% (95% CI 59.0–75.3) when compared to the PCT, 46.7% (95% CI 37.9–55.7) (p = 0.002) (Fig 6B). No significant difference was detected at the 1 hour and 24 hour stages (p <0.05) with both PCT and FCT producing >70% morality after 24 hours (Fig 6B).

## Residual Efficacy on Skin

**Fig 7:**
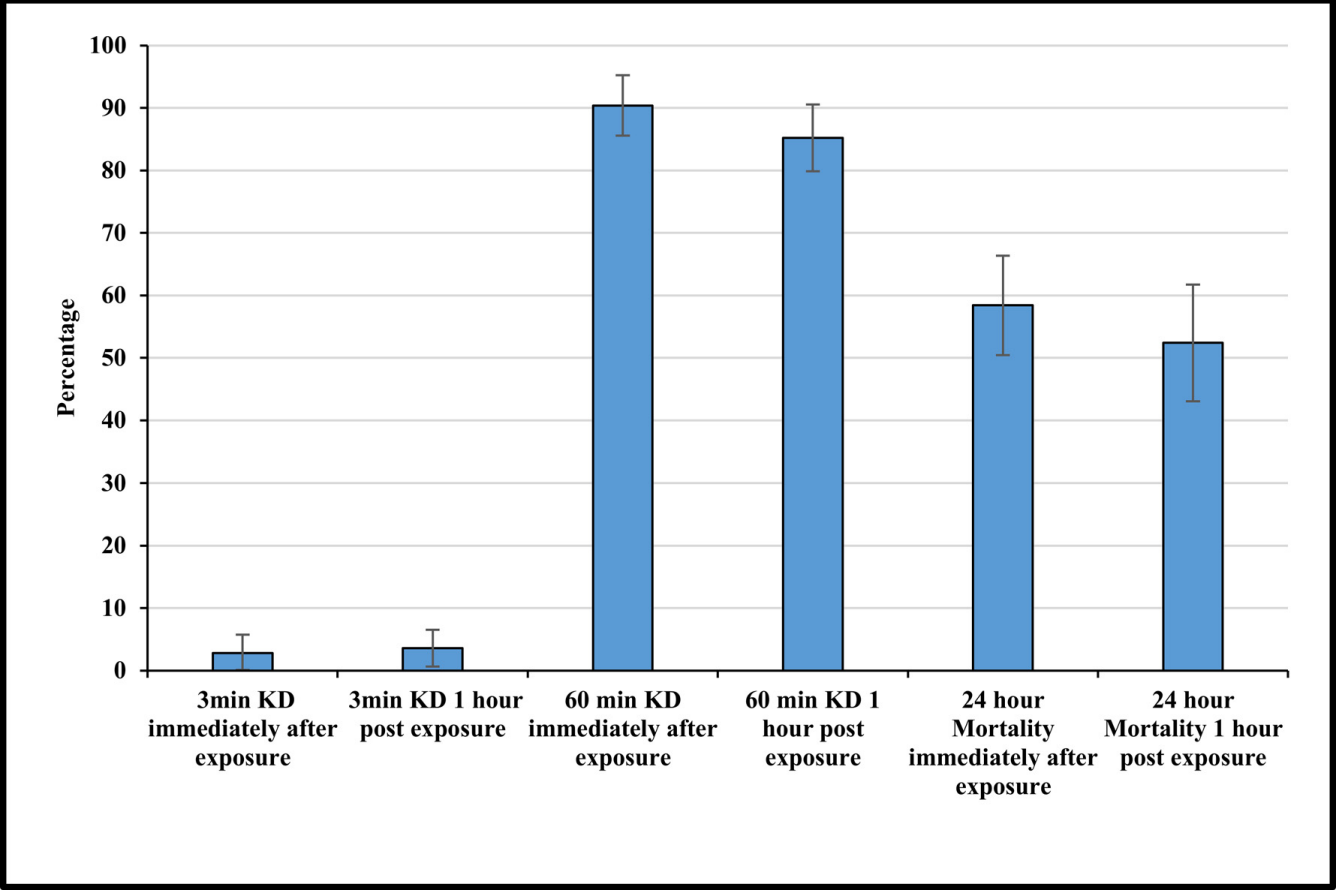
Knockdown after 3 minutes, 1 hour and mortality after 24 hours for mosquitoes exposed to a bare arm, immediately and 1 hour after wearing treated clothing. Whiskers correspond to 95% confidence intervals.

There were no differences in KD or mortality of mosquitoes exposed to skin immediately after removing the treated cloth or 1 hour later (p<0.05) (Fig 7). KD after 3 minutes was 2.8% (95% CI 0.l%–5.7%) when measured immediately after exposure and 3.6% (95% CI 0.6%–6.6%) when measured 1 hour after exposure (Fig 7). Knockdown significantly increased (p = <0.0001) after 1 hour with a KD of 90.4% (95% CI 85.6%–95.2%) and 85.2% (95% CI 79.9%–90.5%) (Fig 7). Mortality after 24 hours was shown to significantly decrease compared to KD after 1 hour (p = <0.0001). Mortality for immediate exposure was 58.4% (95% CI 50.4–66.4%) and for 1 hour post exposure 52.4% (95% CI 43.1%–61.7%) (Fig 7).

**Fig 8:**
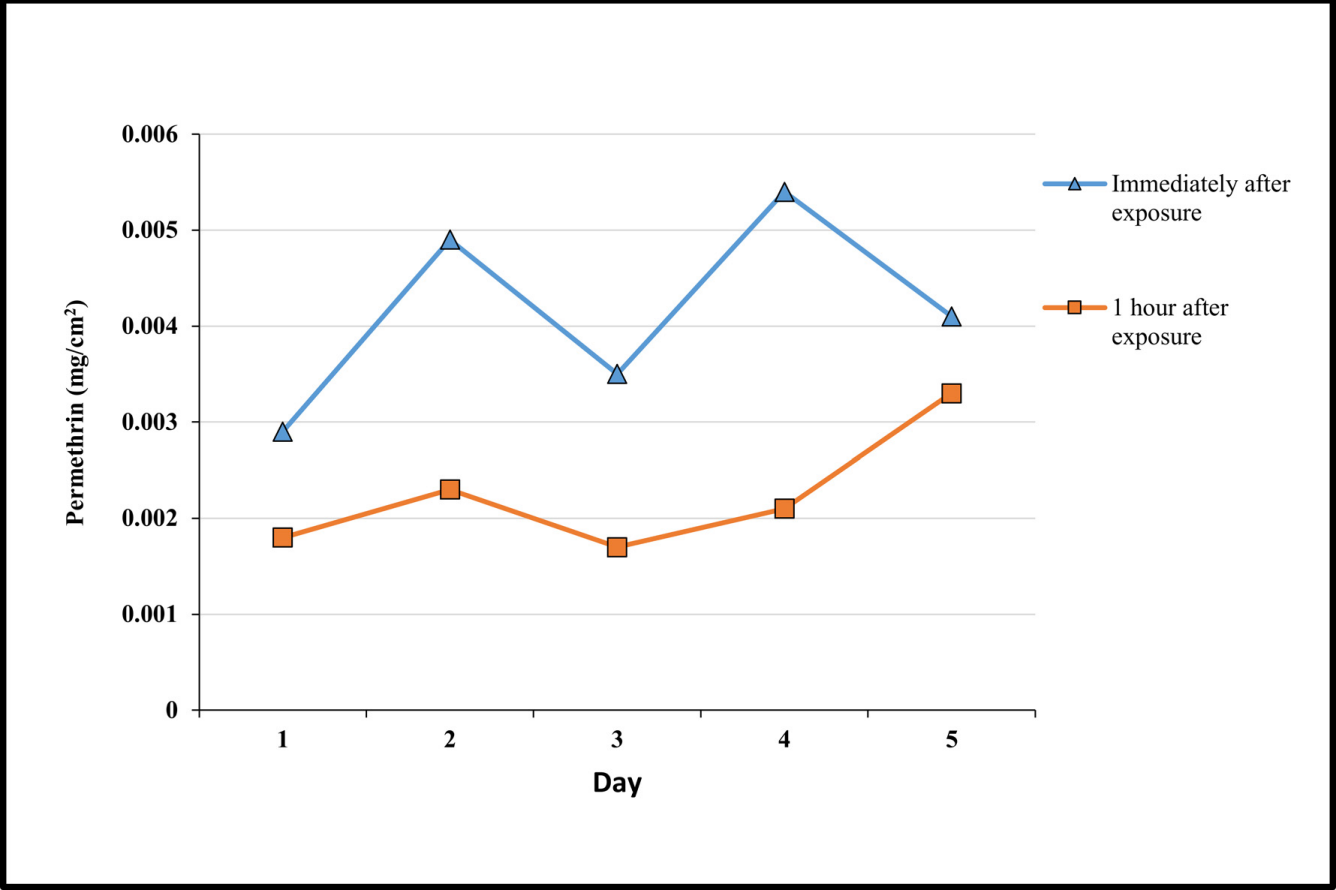
Permethrin concentration of skin swabs taken from individuals wearing clothing immediately after wearing the clothing and 1 hour after wearing the clothing across 5 days.

HPLC showed residual permethrin was present on the skin both immediately and 1 hour after wearing treated clothing (Fig 8). The amount of permethrin decreased 1 hour post exposure. An increase in permethrin was observed as the number of days increased from 0.0029 to 0.0041 mg/ml and 0.0018 to 0.0033 mg/ml for immediate exposure and 1 hour post exposure to the clothing respectively (Fig 8).

## Discussion

Here we have demonstrated that permethrin-treated clothing provides significant protection against landing and biting from *Ae*. *aegypti* mosquitoes and has a significant effect on KD and mortality. In free-flight room tests, permethrin-treated clothing provided the greatest protection against *Ae*. *aegypti* when worn as long-sleeved shirts and long trousers (full coverage) preventing 91% of biting. Treated short-sleeved shirts worn with shorts (partial coverage) were less effective at reducing biting but still achieved 49% protection. Interestingly there was no difference between full and partial coverage of treated clothing in the number of mosquitoes landing on a participant. In both cases, landing was reduced by around 25%.

Besides mechanical protection provided by the clothing, we believe the additional significant reduction in biting observed in our study was due to permethrin affecting mosquito motor skills. Type I pyrethroids such as permethrin affect the voltage gated sodium channels involved in nerve impulses, resulting in repeated firing of neurons [33–35]. This supports our observations, with mosquitoes exhibiting laboured movement, shaking and eventually falling off the arm when in prolonged contact with the clothing. The mosquitoes also demonstrated the characteristic ‘hot feet’ [24] where mosquitoes lift their legs constantly as if standing on a hot surface and are unable to land for a prolonged period of time. As well as its insecticidal properties, permethrin is known to have an “excito-repellency” effect [36–40]. This effect has been observed previously when testing ITN’s and LLINs treated with permethrin, where mosquitoes have demonstrated reduced landing attempts and increased flying activity [41–43]. The protection observed in our study against resistant mosquitoes may also be explained by the excito-repellent effect. For example, when exposed to the partial coverage control (PCC) sleeve, susceptible mosquitoes attempted to probe through the fabric, although unsuccessfully. However, when the susceptible mosquitoes were exposed to the partial coverage treated (PCT) clothing, they appeared to be repelled from the sleeve and were more likely to land on the exposed arm which resulted in successful biting. In contrast, the resistant strain was more likely to land on the treated sleeve and stay there with the sleeve itself providing mechanical protection therefore reducing overall the number of bites received. Permethrin resistant mosquitoes have been shown previously to be less repelled by permethrin than susceptible mosquitoes and will land and stay in contact with permethrin treated materials for longer periods of time [44, 45]. Similarly, studies investigating the efficacy of bed nets in areas of high knockdown resistance (*kdr*) have shown nets treated with permethrin remained effective at killing and reducing biting rates. This is potentially due to resistant mosquitoes landing and staying on treated nets for longer resulting in increased exposure to permethrin and a reduction in biting rates [46, 47]. The above effects could suggest an unexpected benefit of wearing permethrin-treated clothing in regions with pyrethroid-resistant vectors.

Although significant protection was achieved in the free-flight room tests with partial coverage, this may be enhanced by combining permethrin with a repellent active ingredient. Previous studies have demonstrated that bite protection can be greatly increased when insecticide-treated clothing is used in combination with a topical repellent such as *N*,*N*-diethyl-meta-toluamide (DEET) [26, 48, 49] and more recently by incorporating DEET into fabrics [50]. It should also be noted that as well as DEET, there are many potential repellent compounds that could be tested and incorporated into fabric to increase protection [51, 52]. However, the addition of a volatile repellent would require a treatment technique where the repellent remains in the clothing for a substantial period of time.

As well as personal protection, the additional benefit of permethrin-treated clothing is its ability to knockdown and kill mosquitoes during, and even after wearing the clothing. The 70% mortality achieved by the clothing after 24 hours could have a significant effect on the transmission of pathogens that cause diseases like dengue fever and Zika. Previous studies have shown that mosquito feeding behaviour increases when infected with the dengue viruses [53–55]. However, since the dengue virus has an average incubation period of 4.7–6.5 days before mosquitoes are infective [56], the rapid knockdown and mortality caused by permethrin-treated clothing could have an effect before these behavioural changes can occur. Additionally, high knockdown and mortality could aid in the reduction of local vector populations, which in turn could have an effect on the prevalence of disease transmission at the community level. These effects have been demonstrated previously [29, 57, 58] with a significant reduction in malaria transmission with treated clothing and bedding [27–29], but this has not yet been investigated for diseases like dengue and Zika.

Although toxicity of permethrin was not directly measured in this study, multiple studies have shown that skin absorption of permethrin from treated clothing is significantly below WHO and U.S environmental protection agency (EPA) [59–61] maximum recommendations with factory dipped clothing showing the lowest absorption rate of all treatment techniques [62]. Here the residual permethrin detected on the skin was low. However, the levels detected on the skin were enough to produce high knockdown and mortality even one hour after wearing the clothing. This could be beneficial when used in the field, as it implies that there may still be personal protection for some time after the clothing has been removed.

The rapid decrease in efficacy and protection provided by insecticide treated material after washing has been demonstrated previously [63–67]. This could be due to the treatment technique used in this study. As the factory dipping technique only coats the surface of clothing material [62] permethrin could have been rapidly lost during the washing process, resulting in protection decreasing almost 3-fold after just 10 washes during arm-in-cage tests. This suggests that clothing would need to be re-treated or replaced after just 5 machine washes. If clothing was to be used in the longer term in the field, there is an additional risk of exposing mosquitoes to a sub-lethal dose of permethrin which may result in selecting for insecticide resistance in the mosquito population and thus outweighing any benefit of protection provided by the clothing in areas of permethrin resistance. However, this has never been investigated for clothing.

The reduction in efficacy highlights the need for development of clothing with a greater duration of protection. Treatment techniques such as micro-encapsulation, which binds deeper within the fibres of the fabric, allows for increased insecticide stability and a longer lasting release of insecticide [68]. Therefore, clothing treated using the micro-encapsulation technique could provide a longer lasting protection, although this needs to be investigated.

Robust field trials are required to determine the effect of insecticide-treated clothing on disease transmission. Current studies target small populations [29], and school children [69] at high risk of malaria and dengue respectively. However, it would also be beneficial to determine if permethrin-treated clothing could have a significant effect on other mosquito borne disease such as Zika and Chikungunya (also transmitted by *Aedes* mosquitoes), given their recent outbreaks in the Caribbean [70, 71] as well as Central and South America [4, 72, 73].

## Conclusion

Permethrin-treated clothing was shown to be protective against biting by *Ae*. *aegypti* and could have potential as a supplementary tool in the fight against arthropod borne disease such as dengue fever and Zika. The ability for the clothing to reduce biting as well as produce a high knockdown and mortality could have an effect on reducing the risk of dengue and Zika. Although it is important to wear permethrin-treated clothing over as much as the body as possible, the 49% protection provided by the partial coverage treated clothing is promising. It is important that if clothing is implemented in a control program, the efficacy of the clothing remains high for long periods so protection is maintained. Therefore, if permethrin-treated clothing is to have a substantial long-term impact, factors which have a significant effect on the efficacy and duration of protection such as the durability of impregnation after repeated washing need to be considered and treatment techniques further investigated. Despite this, if these issues can be addressed and overcome, permethrin-treated clothing could be a valuable tool in the fight against dengue fever and Zika.
